# Classical swine fever virus utilizes stearoyl-CoA desaturase 1-mediated lipid metabolism to facilitate viral replication

**DOI:** 10.1128/jvi.00551-25

**Published:** 2025-05-19

**Authors:** Ji-shan Bai, Lin-Ke Zou, Ya-Yun Liu, Lin-Han Zhong, Jing Chen, Jin-Xia Chen, Bing-Qian Zhao, Rong-Chao Liu, Bo-Tao Sun, Bin Zhou

**Affiliations:** 1 MOE Joint International Research Laboratory of Animal Health and Food Safety, College of Veterinary Medicine, Nanjing Agricultural University 261674, Nanjing, China; 2 Key Laboratory of Animal Bacteriology, Ministry of Agriculture and Rural Affairs, Nanjing Agricultural University 70578, Nanjing, China; 3 Institute of Animal Husbandry and Veterinary Science, Livestock and Poultry Epidemic Diseases Research Center of Anhui Province, Anhui Province Key Laboratory of Livestock and Poultry Product Safety Engineering, Anhui Academy of Agricultural Sciences, Hefei, China; University of Kentucky College of Medicine, Lexington, Kentucky, USA

**Keywords:** classical swine fever virus (CSFV), stearoyl-CoA desaturase 1 (SCD1), lipid metabolism, IRE1α/XBP1

## Abstract

Viral infections can significantly alter cellular lipid metabolism by modulating key rate-limiting enzymes, including fatty acid synthase (FASN), stearoyl-CoA desaturase 1 (SCD1), and acetyl-CoA carboxylase (ACC). Our previous study revealed the pivotal role of FASN in lipid droplet (LD) synthesis and the promotion of classical swine fever virus (CSFV) replication. However, the roles of the other two key enzymes in CSFV infection remain unexplored. In this study, we screened a library of 96 lipid metabolism-targeted compounds and identified an antiviral inhibitor of SCD1, a rate-limiting enzyme in monounsaturated fatty acid synthesis, that inhibits CSFV replication. Suppressing SCD1 activity through inhibitors or small interfering RNA knockdown reduces CSFV proliferation. However, this suppression is reversed by adding SCD1 active products (oleic acid/palmitoleic acid [OA/PA]), highlighting the essential role of SCD1 in CSFV proliferation. Mechanistically, CSFV non-structural protein p7 interacts with SCD1 and recruits it to the viral replication complex (VRC) during infection. Importantly, CSFV infection activates the endoplasmic reticulum stress pathway IRE1α/XBP1, which positively regulates SCD1 expression, leading to increased production of triglyceride (TG) and LDs and subsequently enhancing CSFV replication. In summary, our study elucidates the critical role of SCD1 in the CSFV life cycle and highlights its potential as an antiviral target for developing new therapies against *Flaviviridae*.

**IMPORTANCE:**

Understanding the virus’s pathogenesis within the host is essential for advancing antiviral therapeutics and vaccine development. Previous studies have demonstrated that classical swine fever virus (CSFV) leverages host lipid metabolic rate-limiting enzymes, such as fatty acid synthase (FASN), to support viral replication. This study identified stearoyl-CoA desaturase 1 (SCD1), a key enzyme in monounsaturated fatty acid biosynthesis, as a novel regulator of CSFV replication. Mechanistically, the viral non-structural protein p7 mediates the recruitment of SCD1 to the endoplasmic reticulum (ER), facilitating the formation of viral replication complexes (VRCs). Additionally, our findings showed that viral infection activated the ER stress pathway IRE1α/XBP1, which upregulated SCD1 expression and promoted the synthesis of triglycerides (TG) and lipid droplets (LDs). This study provides insights into the metabolic reprogramming triggered by viral infection to support replication and underscores the intricate crosstalk between ER stress and lipid metabolism during CSFV infection. These findings have significant implications for identifying novel antiviral targets against CSFV.

## INTRODUCTION

Classical swine fever (CSF) is a highly contagious and lethal infectious disease caused by the classical swine fever virus (CSFV) in domestic and wild pigs, leading to substantial economic losses in the global pig farming industry (1, 2). CSFV, an enveloped, single-stranded, positive-sense RNA virus (3, 4), is a prominent member of the *Pestivirus* genus within the *Flaviviridae* family. Vaccination remains the primary strategy for preventing and managing CSF in production (5). However, the virulence of CSFV strains, along with factors such as host age and immune status, frequently leads to covert and persistent infections in clinical settings, posing challenges to the prevention and eradication of CSF (6, 7). Therefore, the development and identification of effective antiviral drugs for CSF remain a key focus. Lipids, serving as essential components of cell membranes, signaling molecules, and energy reservoirs, play a pivotal role in cellular functions (8–10). Increasing evidence indicates that the modulation of lipid metabolism by various viruses plays a critical role in their replication cycle and influences viral infection (11). For example, flaviviruses, such as hepatitis C virus (HCV) and dengue virus (DENV), are RNA viruses extensively studied for their regulation of neutral lipid metabolism and lipid droplet (LD) during infection (11–13). Rate-limiting enzymes play a pivotal role in metabolic processes; therefore, viral regulation of lipid metabolism is intrinsically tied to modulating the function or abundance of these enzymes (14). Notably, our previous research has demonstrated that CSFV infection upregulates fatty acid synthase (FASN) expression, while FASN knockdown suppresses CSFV replication. FASN contributes to the assembly of replication complexes linked to the endoplasmic reticulum (ER) and interacts with CSFV NS4B, thereby enhancing its own expression, a process modulated by functional Rab18 (15). Furthermore, the role of FASN in Chikungunya virus (CHIKV) replication has been well-established (16). Moreover, the acetyl-CoA carboxylase (ACC) inhibitor CP640186 effectively suppressed DENV infection by enhancing ACC phosphorylation levels, highlighting a direct association between ACC activity and viral replication capacity (17). Stearoyl-CoA desaturase 1 (SCD1) is a key rate-limiting enzyme in fatty acid metabolism, responsible for catalyzing the conversion of saturated fatty acids into monounsaturated fatty acids (MUFAs) (18). SCD1 participates in various cellular processes, including autophagy (19, 20), by promoting autophagosome formation, regulating autophagy via lipid biosynthesis, and influencing autophagosome-lysosome fusion (21, 22). Furthermore, the role of SCD1 in viral infection is increasingly understood. Early studies have revealed that SCD1 facilitates the enrichment of HCV NS5A and NS4B proteins on detergent-resistant membranes, while SCD1 knockdown impairs NS4B-induced membrane rearrangement, a critical step in HCV replication complex formation (23). SCD1 is essential for DENV2 replication, with its enzymatic activity being critical for viral genome replication and particle release (24). Moreover, SCD1 regulates foot-and-mouth disease virus (FMDV) replication by modulating host lipid metabolism and facilitating the assembly of the viral replication complex (VRC) mediated by FMDV non-structural protein 2c (25). Additionally, previous studies have indicated that SCD1 influences the replication of other RNA viruses, such as respiratory enteric orphan virus-3-176, poliovirus-1, enterovirus 71, and vesicular stomatitis virus. Nevertheless, the role of SCD1 in CSFV infection remains unexplored. This study uncovers a novel role of SCD1 in CSFV replication. In this study, SCD1, a key metabolic rate-limiting enzyme, was targeted by inhibitors that suppressed the replication of CSFV as well as bovine viral diarrhea virus (BVDV), Japanese encephalitis virus (JEV), and pseudorabies virus (PRV), highlighting SCD1 as a potential broad-spectrum antiviral target. SCD1 interacted with the CSFV non-structural protein p7 and was recruited to the VRC. During CSFV infection, SCD1 was modulated by the ER stress pathway IRE1α/XBP1 and upregulated the levels of triglyceride (TG) and LDs. Overall, our findings elucidate the pivotal role of SCD1 in CSFV infection, advance the understanding of SCD1 in viral research, and offer valuable insights for antiviral drug development.

## RESULTS

### SCD1 is an effective target for CSFV

**Fig 1:**
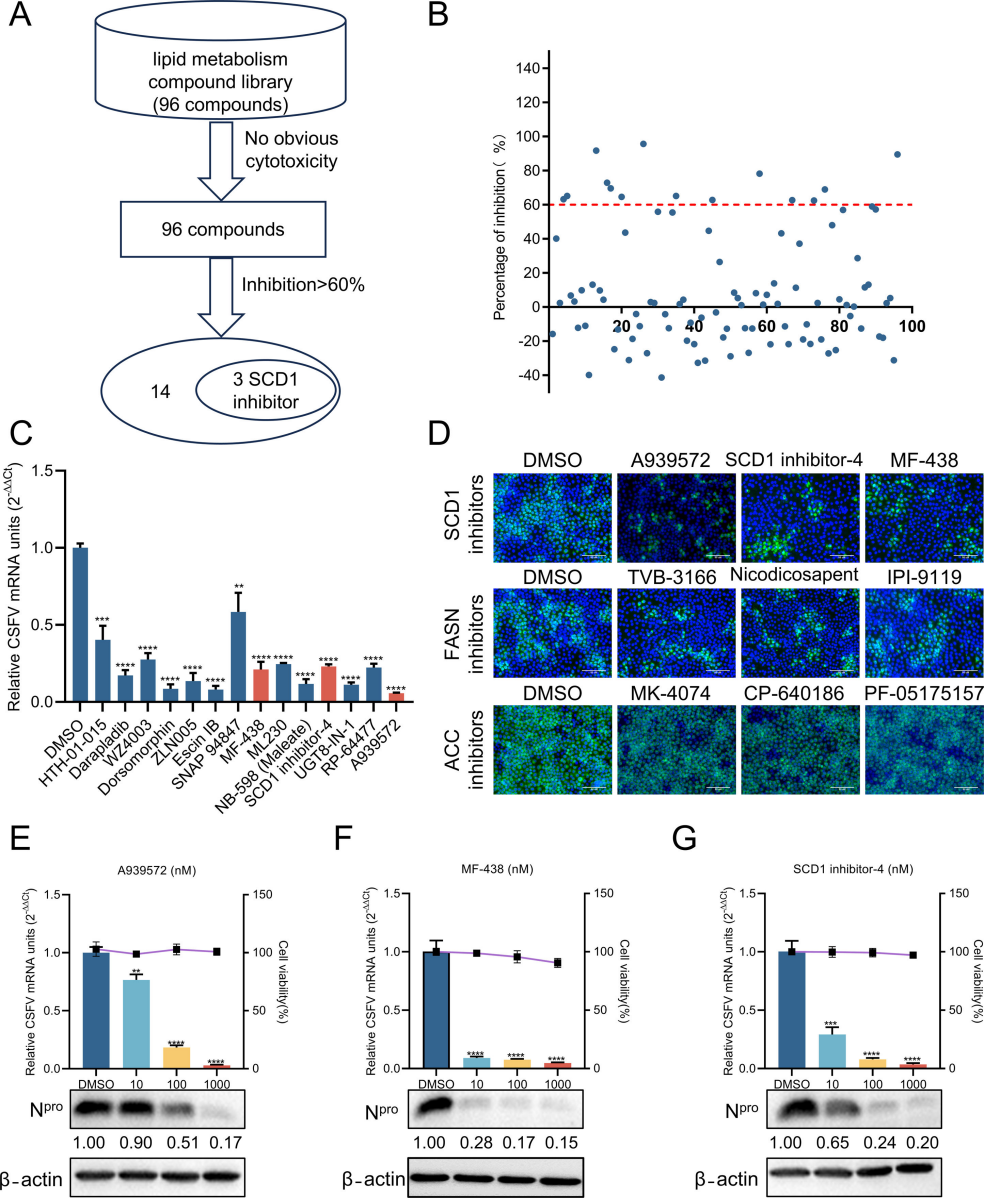
Screening of anti-CSFV compounds from a lipid metabolism library. (**A**) Screening process for antiviral compounds. (**B**) Inhibition effects of the compounds. Each dot represents the inhibition rate of a compound (2 µM) against CSFV (MOI = 1). (**C**) PK-15 cells infected with CSFV (MOI = 1) for 24 h were collected and analyzed by RT-qPCR to measure CSFV mRNA levels. (**D**) Antiviral effects of compounds targeting three key rate-limiting enzymes (SCD1, FASN, and ACC) in lipid metabolism. PK-15 cells infected with CSFV (MOI = 1) were treated with inhibitors of rate-limiting enzymes, then fixed and stained with anti-CSFV E2 antibody (green) for fluorescence microscopy. Scale bars = 50 µm. (E through G) Antiviral effects of three SCD1-targeting compounds. PK-15 cells were treated with DMSO or varying concentrations of A939572, MF-438, and SCD1 inhibitor-4 for 24 h after infection with CSFV (MOI = 1). Viral replication was assessed by Western blotting and RT-qPCR. Cell cytotoxicity was quantified using the CCK-8 assay. β-Actin was used as a loading control, and protein expression levels were calculated by determining the ratio of the protein to β-actin using ImageJ 7.0 software. Data are presented as the mean ± SD from three independent experiments. **P < 0.01, ***P < 0.001, ****P < 0.0001.

Viral infection triggers a series of metabolic changes in the host cell, resulting in metabolic reprogramming, including lipid, nucleotide, and glucose metabolism (26–28). Lipid metabolism plays a vital role in cellular processes, functioning not only as an energy source, but also as signaling molecules regulating the cell cycle, development, antiviral responses, and apoptosis (29). To investigate the influence of lipid metabolism on CSFV replication, a library of 96 lipid metabolism-related compounds was screened on PK-15 cells using an indirect immunofluorescence assay (IFA). Fourteen compounds were identified that exhibited significant inhibitory rates exceeding 60% against CSFV proliferation, three of which were SCD1 inhibitors (Fig. 1A through C). Consequently, we concentrated on SCD1 inhibitors. As a positive control, compounds targeting FASN exhibited significant inhibitory effects on CSFV proliferation (Fig. 1D), consistent with previous findings (15). Unexpectedly, inhibitors targeting ACC, a key rate-limiting enzyme in lipid metabolism, did not exhibit significant effects on CSFV proliferation during this screening process (Fig. 1D). We hypothesized that the drug concentration used in the screening was not optimal for ACC inhibitors, or that ACC played a negligible role in CSFV infection. To evaluate the inhibitory effects of A939572, MF-438, and SCD1 inhibitor-4 on CSFV replication, PK-15 cells were infected with CSFV (multiplicity of infection [MOI] = 1) and treated with varying concentrations of the three compounds for 24 h. The results demonstrated that A939572, MF-438, and SCD1 inhibitor-4 significantly inhibited CSFV replication in a dose-dependent manner at both transcriptional and translational levels, without compromising cell viability. Detailed data revealed that, compared to the control group, A939572, MF-438, and SCD1 inhibitor-4 reduced CSFV N^pro^ protein levels by 83.0%, 85.0%, and 80.0%, respectively, and RNA copy numbers by 97.0%, 95.6%, and 96.5%, respectively (Fig. 1E through G). In summary, SCD1 represents a promising target for antiviral therapy.

### SCD1 is a broad-spectrum antiviral target against *Flaviviridae* and porcine herpesvirus type 1

**Fig 2:**
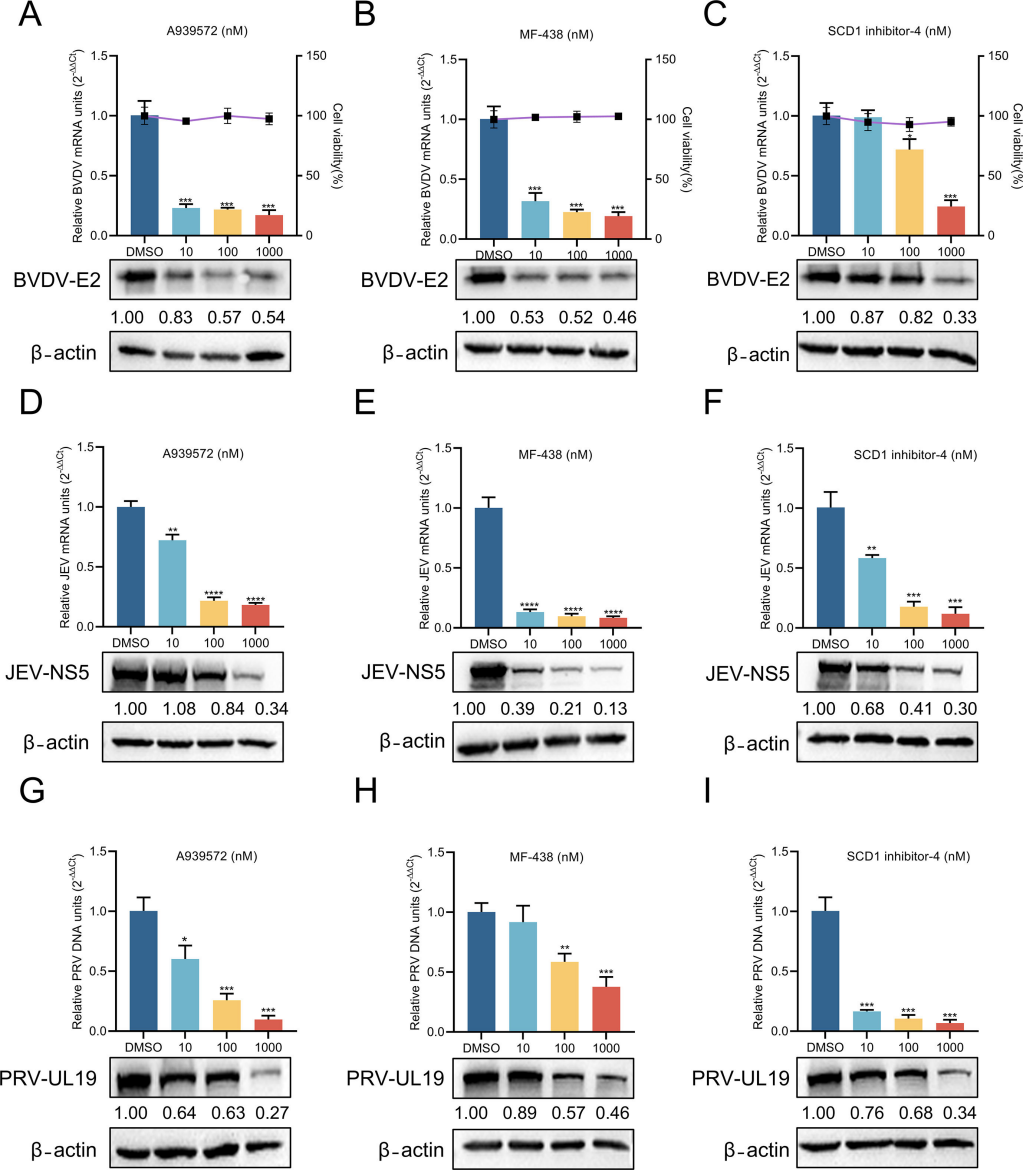
SCD1 is a broad-spectrum antiviral target. (A through C) MDBK cells were treated with DMSO or varying concentrations of A939572, MF-438, or SCD1 inhibitor-4 for 24 h after infection with BVDV (MOI = 0.5). Viral replication was assessed by Western blotting and RT-qPCR. The effects of the indicated compounds on cell cytotoxicity were quantified using the CCK-8 assay. (D through I) PK-15 cells were treated with DMSO or varying concentrations of A939572, MF-438, or SCD1 inhibitor-4 for 24 h after infection with JEV or PRV (MOI = 0.1). Viral replication was assessed by RT-qPCR (JEV) or qPCR (PRV) and Western blotting. β-Actin served as a loading control. Protein expression levels were quantified by calculating the ratio of protein to β-actin using ImageJ 7.0 software. Data are presented as the mean ± SD from three independent experiments. **P < 0.01, ***P < 0.001, ****P < 0.0001.

SCD1 inhibitors have been demonstrated to effectively suppress the replication of diverse RNA viruses, including HCV (13), DENV (30), Zika (31), and FMDV (25). Additionally, we evaluated the *in vitro* inhibitory efficacy of three compounds against other significant animal viruses. The respective cells were infected with two members of the *Flaviviridae* family, including JEV (32) and BVDV (33), as well as PRV (porcine herpesvirus type 1) (34), followed by treatment with varying concentrations of these compounds for 24 h. RT-qPCR and Western blotting analyses demonstrated that the three compounds significantly inhibited the replication of JEV, BVDV, and PRV in a dose-dependent manner. Relative to the control group, A939572, MF-438, and SCD1 inhibitor-4 decreased BVDV E2 protein levels by 46.0%, 54.0%, and 67.0%, respectively, and RNA copy numbers by 82.9%, 80.8%, and 75.6% (Fig. 2A through C). Furthermore, A939572, MF-438, and SCD1 inhibitor-4 exhibited significant inhibitory effects on JEV replication. Specifically, these compounds decreased JEV NS5 protein levels by 56.0%, 87.0%, and 70.0%, respectively, and viral RNA levels by 81.9%, 99.2%, and 99.2% (Fig. 2D through F). Beyond the aforementioned RNA viruses, A939572, MF-438, and SCD1 inhibitor-4 also suppressed PRV replication. These compounds reduced PRV UL19 protein levels by 73.0%, 54.0%, and 66.0%, and viral DNA levels by 90.32%, 62.36%, and 95.59%, respectively (Fig. 2G through I). Collectively, these findings highlight SCD1 as a broad-spectrum antiviral target for combating multiple viruses.

### SCD1 inhibitors play the main effect on the late stage of CSFV infection

**Fig 3:**
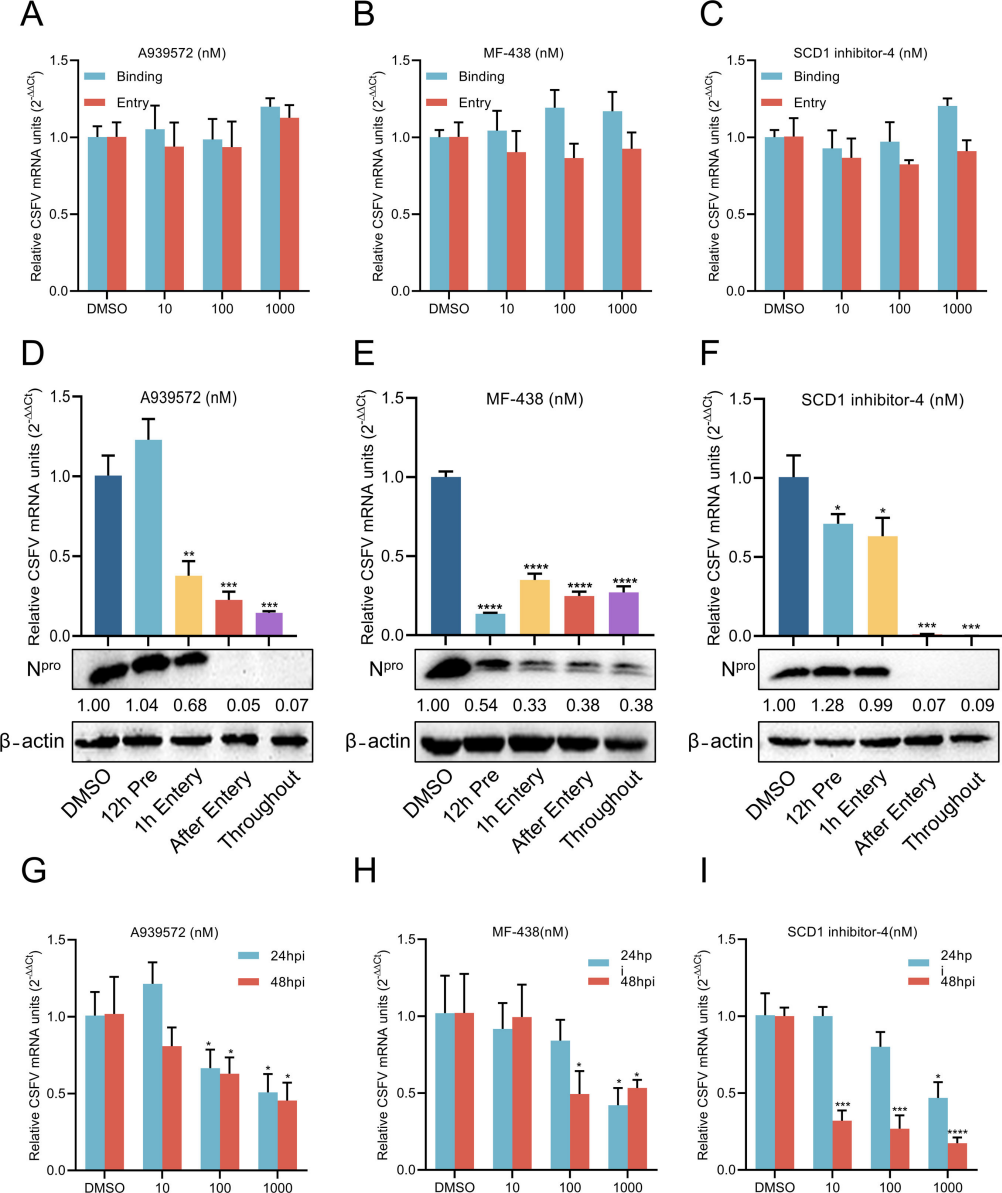
The SCD1 inhibitors mainly affect the late stage of CSFV infection. (A through C) PK-15 cells were treated with varying concentrations of A939572, MF-438, or SCD1 inhibitor-4 and infected with CSFV (MOI = 10) for 1 h at 4°C, followed by incubation at 37°C for either 0 hpi (binding) or 1 hpi (entry). Cells were harvested and analyzed by RT-qPCR to measure viral mRNA levels. (D through F) The drug action at specific stages was evaluated using a time-of-addition assay, as described below. (i) 12 h pretreatment: cells were treated with the indicated concentration (1,000 nM) of A939572, MF-438, or SCD1 inhibitor-4 for 12 h before infection with CSFV (MOI = 1); (ii) 1 h entry: cells were treated with a mixture of the indicated SCD1 inhibitors and CSFV (MOI = 1) for 1 h; (iii) post-entry: cells were treated with the indicated SCD1 inhibitors after infection with CSFV; (iv) throughout: cells were treated with the indicated SCD1 inhibitors throughout the entire process. Treated cells were collected at 24 hpi and analyzed by RT-qPCR and Western blotting to assess viral replication. β-Actin served as a loading control. Protein expression levels were quantified by calculating the ratio of protein to β-actin using ImageJ 7.0 software. (G through I) Time-dependent antiviral effects. Cells infected with CSFV (MOI = 1) were treated with varying concentrations of A939572, MF-438, or SCD1 inhibitor-4 for the indicated time, followed by collection of the supernatant to infect fresh cells for an additional 24 h. Samples were collected and subjected to RT-qPCR for detecting viral mRNA levels. Data are presented as the mean ± SD from three independent experiments. *P < 0.05, **P < 0.01, ***P < 0.001, ****P < 0.0001.

To determine whether SCD1 inhibitors affect the early stages of the CSFV life cycle, we examined their impacts on CSFV binding and entry into target cells. Pretreatment cells with A939572, MF-438, or SCD1 inhibitor-4 did not reduce CSFV binding or entry (Fig. 3A through C). To further elucidate the mechanisms of action, time-of-addition assays were conducted, with 1,000 nM A939572, MF-438, and SCD1 inhibitor-4 added to cells before or during CSFV infection. A slight reduction in viral mRNA levels was observed in cells pretreated with SCD1 inhibitor-4 for 12 h, with no significant changes in protein levels. In contrast, cells treated with A939572 showed no significant differences in viral mRNA production or protein expression. Administering A939572 and SCD1 inhibitor-4 1 h post-virus entry resulted in a slight reduction in viral mRNA levels. A marginal decrease in protein levels was observed with A939572, while SCD1 inhibitor-4 showed no significant effect on protein levels. Delayed addition of A939572 and SCD1 inhibitor-4, 1 hpi and maintained for the infection duration (24 h post-entry), significantly reduced both viral mRNA production and protein expression. However, MF-438 demonstrated inhibitory effects across all stages of the CSFV life cycle (Fig. 3D through F). These findings suggest that A939572 and SCD1 inhibitor-4 primarily affect the later stages of CSFV infection, whereas MF-438 impacts all stages of the viral life cycle. Notably, a greater reduction in virus production was observed when cells were treated with A939572 or SCD1 inhibitor-4 12 h prior to infection and maintained throughout the infection period. Furthermore, to assess whether SCD1 inhibitors influence CSFV release, cells were infected with CSFV and treated with A939572, MF-438, or SCD1 inhibitor-4 for 24 and 48 h. The cell supernatants were then collected to infect fresh cells for an additional 24 h. Quantitative viral genome analysis identified a significant reduction in viral RNA (Fig. 3G through I). Collectively, these findings indicate that A939572, MF-438, and SCD1 inhibitor-4 primarily exert their effects during the replication phase rather than the entry phase of the CSFV life cycle.

### CSFV replication requires SCD1

**Fig 4:**
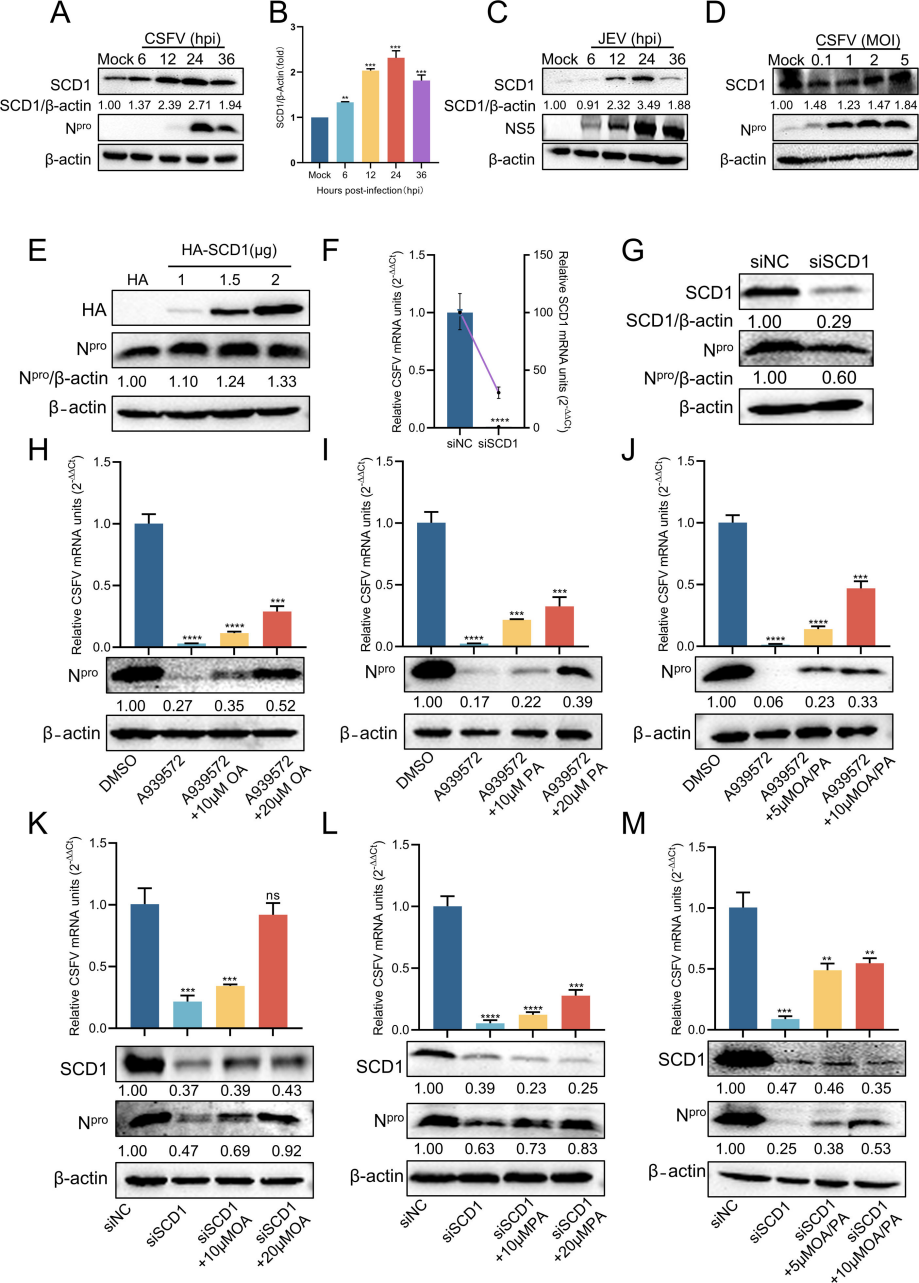
SCD1 positively regulates CSFV replication. (**A**) PK-15 cells infected with CSFV (MOI = 1) were collected at the indicated time points and analyzed by Western blotting using rabbit anti-CSFV N^pro^ antibody. (**B**) The SCD1 content shown in panel A was analyzed through grayscale analysis using ImageJ software. (**C**) BHK-21 cells infected with JEV (MOI = 0.1) were collected at the indicated time points and analyzed by Western blotting using mouse anti-JEV NS5 antibody. (**D**) PK-15 cells infected with varying MOIs of CSFV were collected at the indicated time points and analyzed by Western blotting. (E) PK-15 cells transfected with pHA-SCD1 (1, 1.5, and 2 µg) were infected with CSFV (MOI = 1). At 24 hpi, cells were collected and analyzed by Western blotting. (F and G) PK-15 cells transfected with siNC or siSCD1 for 24 h were infected with CSFV (MOI = 1). Cells were collected and analyzed by RT-qPCR and Western blotting. (H through J) PK-15 cells infected with CSFV (MOI = 1) were treated with A939572 (1 µM) and varying concentrations of OA/PA in 2% DMEM for 24 h. Treated cells were collected and analyzed by RT-qPCR and Western blotting. (K through M) PK-15 cells transfected with siSCD1 for 24 h were infected with CSFV (MOI = 1) and treated with varying concentrations of OA/PA in 2% DMEM for 24 h. Treated cells were collected and analyzed by RT-qPCR and Western blotting. β-Actin served as a loading control. Protein expression levels were quantified by calculating the ratio of protein to β-actin using ImageJ 7.0 software. Data are presented as the mean ± SD from three independent experiments. **P < 0.01, ***P < 0.001, ****P < 0.0001.

Our studies substantiated that SCD1 was an effective antiviral target for CSFV. To investigate the role of SCD1 in CSFV infection, PK-15 cells were infected with CSFV (MOI = 1) and harvested at specified time points for Western blotting analysis. The results indicated that SCD1 protein expression increased over time, peaked at 24 hpi, and then gradually decreased (Fig. 4A). Statistical analyses further corroborated these findings, demonstrating that CSFV infection significantly upregulates SCD1 protein levels (Fig. 4B). This result is consistent with the trend of SCD1 upon JEV infection (Fig. 4C). Additionally, cells infected with CSFV at varying MOIs showed that higher MOIs correlated with increased SCD1 protein levels (Fig. 4D). Next, cells were transfected with varying concentrations of pHA-SCD1 plasmids and infected with CSFV. At 24 hpi, Western blotting analysis revealed that CSFV replication augmented in proportion to increasing HA-SCD1 protein levels (Fig. 4E). Ultimately, the attenuation of endogenous SCD1 expression was achieved through small interfering RNA (siRNA) transfection, which markedly inhibited CSFV proliferation, as evidenced by RT-qPCR and Western blotting analyses (Fig. 4F and G). A complementation experiment was conducted to further substantiate our findings. Infected cells were treated with the SCD1 inhibitor A939572, subsequently treated with oleic acid (OA), palmitoleic acid (PA), or a combination of both. At 24 hpi, RT-qPCR and Western blotting analyses revealed that the addition of OA, PA, or both partially restored CSFV replication (Fig. 4H through J). Similarly, in SCD1 knockdown cells, complementation with OA or PA also restored CSFV replication (Fig. 4K through M). Overall, we have established that SCD1 is indispensable for CSFV proliferation, and the replication inhibition induced by A939572 or siRNA knockdown can be partially ameliorated through exogenous supplementation with OA and PA.

### SCD1 is recruited to the viral replication complex by CSFV p7

**Fig 5:**
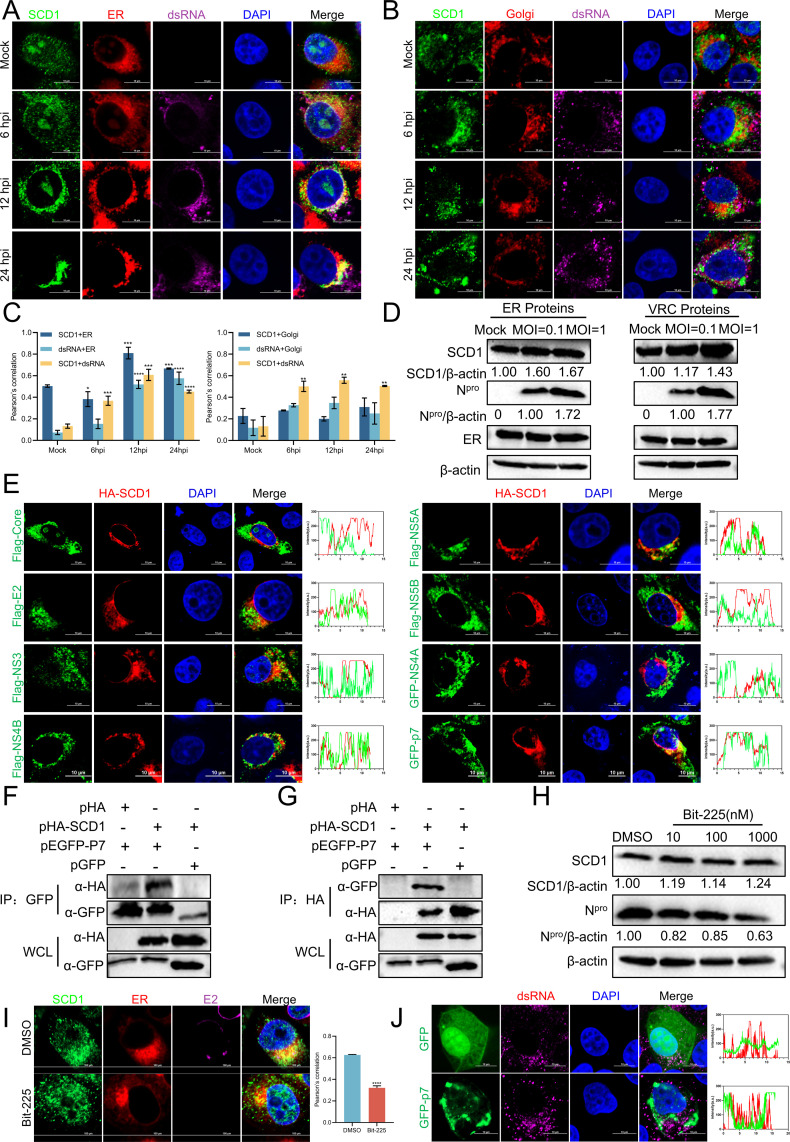
SCD1 is recruited to the viral replication complex by CSFV p7. (**A**) PK-15 cells infected with CSFV (MOI = 1) for 24 h were fixed and stained with mouse anti-dsRNA monoclonal antibody (J2), ER-Tracker Red dye, and rabbit anti-SCD1 antibody for confocal microscopy. Scale bars = 10 µm. (**B**) PK-15 cells infected with CSFV (MOI = 1) for 24 h were fixed and stained with mouse anti-dsRNA monoclonal antibody (J2), Golgi-Tracker Red dye, and rabbit anti-SCD1 antibody for confocal microscopy. Scale bars = 10 µm. (**C**) Co-localization analysis of panels (A, B) was performed using Pearson’s correlation coefficient. (**D**) PK-15 cells infected with CSFV for 24 h were collected, and ER/VRC proteins were extracted using a commercial kit. Western blotting was performed to determine the relative levels of SCD1 protein. (**E**) PK-15 cells were co-transfected with pHA-SCD1 and different CSFV plasmids for 24 h. Cells were then fixed and stained with appropriate antibodies for confocal microscopy. Scale bars = 10 µm. Co-localization analysis was performed using ImageJ. (**F, G**) 293T cells were co-transfected with pHA-SCD1 and pGFP-SCD1 for 24 h, harvested for Co-IP using anti-GFP/HA antibodies, and the resulting proteins were analyzed by Western blotting with specific antibodies. (**H**) PK-15 cells were treated with DMSO or varying concentrations of Bit-225 for 24 h after infection with CSFV (MOI = 1). Viral replication was assessed by Western blotting. β-Actin served as a loading control. (**I**) PK-15 cells infected with CSFV (MOI = 1) for 24 h were fixed and stained with rabbit anti-SCD1 antibody, ER-Tracker Red dye, and mouse anti-E2 antibody for confocal microscopy. Scale bars = 100 µm. (**J**) PK-15 cells were transfected with pGFP, pGFP-p7, then cells infected with CSFV (MOI = 1) for 24 h were fixed and stained with mouse anti-dsRNA monoclonal antibody (J2) for confocal microscopy. Scale bars = 100 µm. Protein expression levels were quantified by calculating the ratio of protein to β-actin using ImageJ 7.0 software. Data are presented as the mean ± SD from three independent experiments. *P < 0.05, **P < 0.01, ***P < 0.001, ****P < 0.0001.

To investigate the relationship between SCD1 and the viral replication complex, we first examined whether SCD1 co-localized with the CSFV replication complex. Cells were infected with CSFV and harvested at indicated time points for confocal microscopy. After CSFV infection, starting from 12 hpi, SCD1 formed a triple co-localization with the viral replication complex and ER, and this co-localization was also observed at 24 hpi (Fig. 5A); but no significant co-localization of SCD1 and double-stranded RNA (dsRNA) with the Golgi apparatus was observed (Fig. 5B). Further analysis of the co-localization coefficient further corroborated this phenomenon (Fig. 5C). Additionally, cells were infected with varying MOIs of CSFV, and the total ER proteins and CSFV VRCs were extracted for Western blotting. Results demonstrated that SCD1 expression increased in both the ER and VRC with higher MOIs, suggesting that SCD1 was recruited to the CSFV replication complex (Fig. 5D). To identify the CSFV proteins that are crucial for SCD1 function, PK-15 cells were transfected with indicated constructs overexpressing CSFV structural proteins (Core, E2) and non-structural proteins (NS3, NS4A, NS4B, NS5A, NS5B, p7). The transfected cells were subsequently fixed and stained with specific antibodies for confocal microscopy. Interestingly, SCD1 co-localized with p7 remarkably, but not with Core, E2, NS3, NS4B, NS5A, or NS5B (Fig. 5E). To investigate the interaction between SCD1 and p7, PK-15 cells were co-transfected with constructs pHA-SCD1 and pEGFP-p7 for 24 h, then harvested and lysed for co-immunoprecipitation (Co-IP) assay. The results elucidated a definitive interaction between SCD1 and p7 (Fig. 5F and G). To explore whether p7 mediates the recruitment of SCD1 to the viral replication complex, the ion channel inhibitor Bit-225 was used in this experiment. To assess the inhibitory effects of Bit-225 on CSFV replication, PK-15 cells were infected with CSFV (MOI = 1) and treated with varying concentrations for 24 h. The results showed that Bit-225 significantly inhibited CSFV replication in a dose-dependent manner, without affecting SCD1 protein (Fig. 5H). The confocal microscopy showed that after treatment with the Bit-225 inhibitor, the co-localization of SCD1 with the ER was reduced compared to the control group, indicating that the process of SCD1 recruitment to the VRC was mediated by p7 (Fig. 5I). Additionally, confocal microscopy revealed no co-localization between p7 and CSFV dsRNA, indicating that p7 was not involved in the formation of the CSFV VRC (Fig. 5J). Previous studies have shown that p7 primarily localizes to the ER during the early stage of expression and transitions to the plasma membrane at a later stage (35). Based on these findings, we propose that p7 interacts with SCD1 at the ER and subsequently sequesters SCD1 to transfer it to the CSFV VRC.

### CSFV infection induces the ER stress pathway IRE1α/XBP1 to regulate SCD1

**Fig 6:**
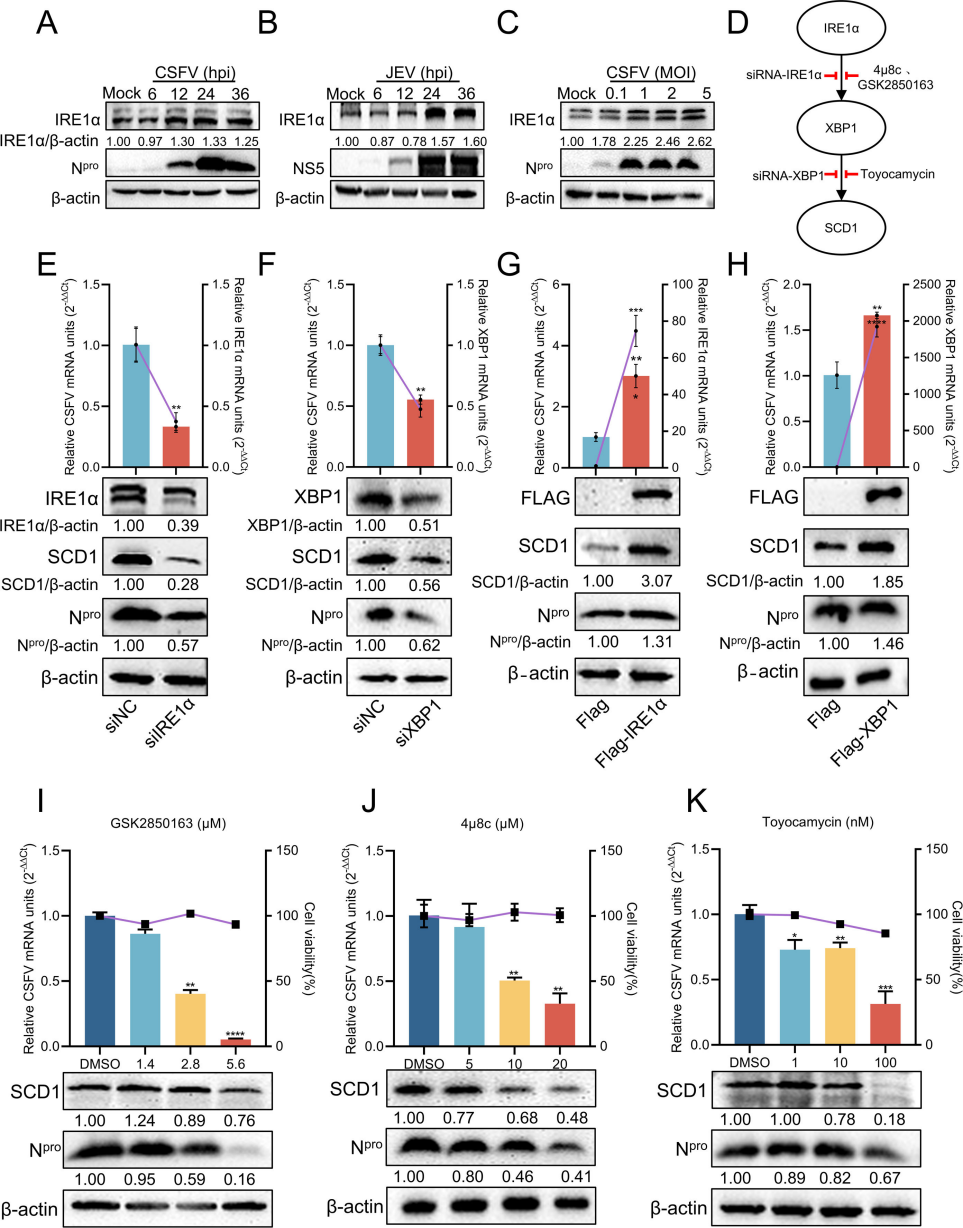
CSFV infection induces the endoplasmic reticulum stress pathway IRE1α/XBP1 to regulate SCD1. (A and B) PK-15 cells infected with CSFV (MOI = 1) or BHK-21 cells infected with JEV (MOI = 0.1) were collected at the indicated time points and analyzed by Western blotting using rabbit anti-CSFV N^pro^, mouse anti-JEV NS5, or rabbit anti-IRE1α antibodies. (**C**) PK-15 cells infected with varying MOIs of CSFV were collected at the indicated time points and analyzed by Western blotting. (**D**) Schematic overview of the IRE1α/XBP1/SCD1 signaling pathway. (E and F) PK-15 cells transfected with siNC, siIRE1α, or siXBP1 for 24 h were infected with CSFV (MOI = 1). Cells were then collected and analyzed by RT-qPCR and Western blotting. (G and H) PK-15 cells transfected with p3×Flag-IRE1α and p3×Flag-XBP1, respectively, and infected with CSFV (MOI = 1) for 24 h. Cells were then collected and analyzed by RT-qPCR and Western blotting. (I through K) PK-15 cells infected with CSFV (MOI = 1) were treated with IRE1α/XBP1 inhibitors for 24 h. Cells were then collected and analyzed by RT-qPCR and Western blotting. PK-15 cells infected with CSFV (MOI = 1) were treated with IRE1α/XBP1 inhibitors for 24 h. Cells were then collected and analyzed by RT-qPCR and Western blotting. β-Actin served as a loading control. Protein expression levels were quantified by calculating the ratio of protein to β-actin using ImageJ 7.0 software. Data are presented as the mean ± SD from three independent experiments. *P < 0.05, **P < 0.01, ***P < 0.001, ****P < 0.0001.

To determine whether CSFV infection induces ER stress, cells were infected with CSFV (MOI = 1) and harvested at specified time points for Western blotting analysis. The results revealed that IRE1α protein expression increased over time during CSFV infection (Fig. 6A), consistent with the trend observed during JEV infection (Fig. 6B). Additionally, cells infected with CSFV at varying MOIs and harvested at 24 hpi showed that higher MOIs correlated with increased IRE1α protein levels (Fig. 6C). To assess whether the IRE1α/XBP1 signaling pathway regulates SCD1 levels, cells were treated with IRE1α/XBP1 pathway inhibitors or specific siRNA, followed by CSFV infection (Fig. 6D). Secondly, knockdown of endogenous IRE1α significantly suppressed CSFV proliferation and reduced SCD1 protein levels (Fig. 6E). Similarly, knockdown of XBP1 significantly inhibited CSFV replication, accompanied by a marked reduction in SCD1 protein levels (Fig. 6F). Furthermore, the successive transfection of p3×Flag-IRE1α and p3×Flag-XBP1 constructs into PK-15 cells, subsequently followed by infection with CSFV (MOI = 1), elucidated that the overexpression of these ER stress-associated proteins not only substantially enhanced viral replication but also resulted in a pronounced upregulation of SCD1 protein expression (Fig. 6G and H). These findings suggest that SCD1 is regulated by the IRE1α/XBP1 signaling pathway. To further validate this, cells were treated with GSK2850163 (IRE1α kinase inhibitor) or 4µ8c (IRE1α RNase inhibitor) and infected with CSFV (MOI = 1). Inhibition of either the kinase or RNase activity of IRE1α suppressed CSFV proliferation, with a corresponding reduction in SCD1 protein expression (Fig. 6I and J). Similarly, treatment with the XBP1 inhibitor toyocamycin suppressed CSFV proliferation and reduced SCD1 protein expression (Fig. 6K). In summary, CSFV infection induces ER stress and regulates SCD1 production via the IRE1α/XBP1 signaling pathway.

### The IRE1α/XBP1/SCD1 axis affects the synthesis of triglyceride and lipid droplets during CSFV infection

**Fig 7:**
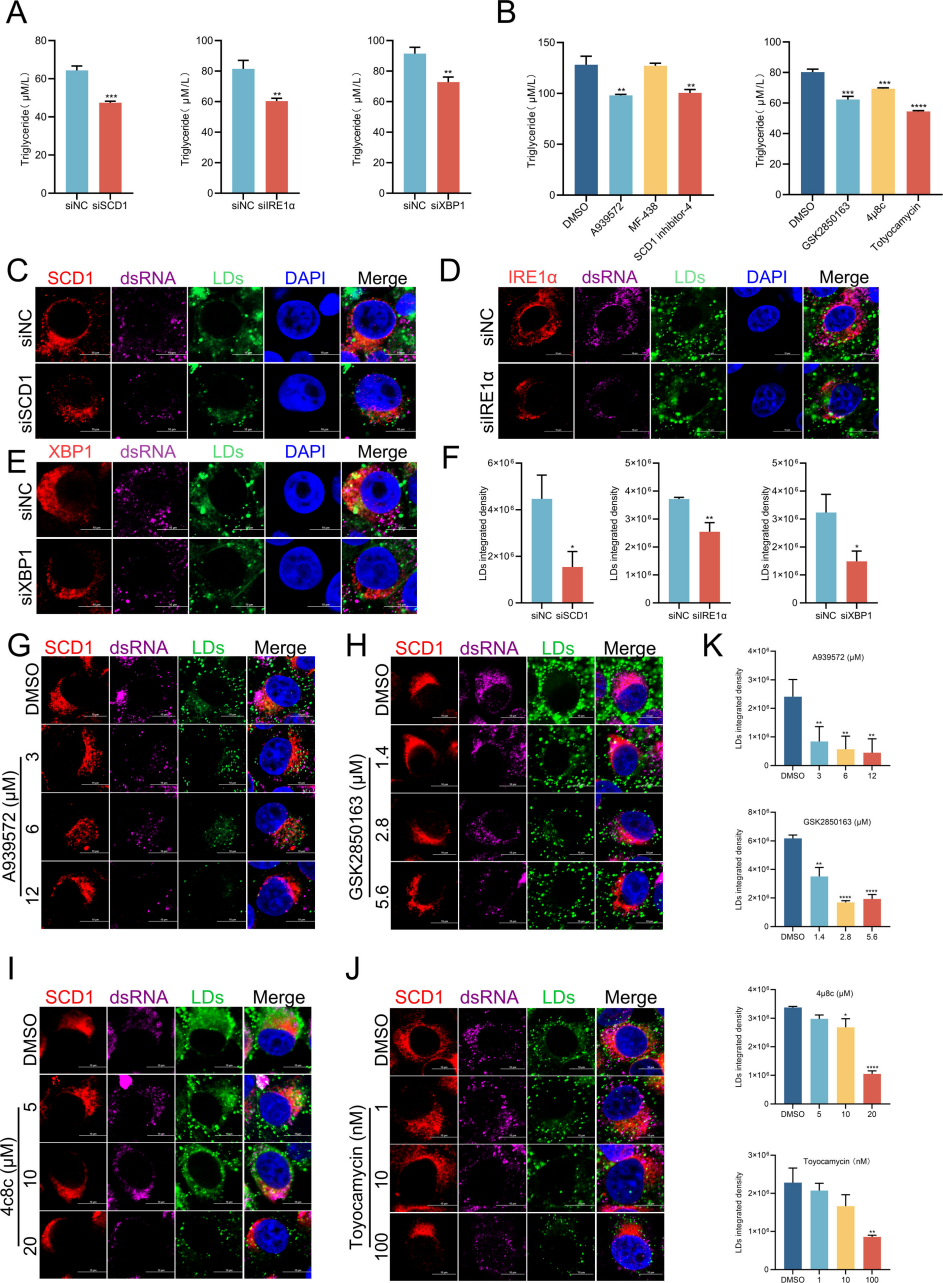
The IRE1α/XBP1/SCD1 axis affects the synthesis of triglyceride and lipid droplets during CSFV infection. (**A**) Triglyceride detection. PK-15 cells transfected with siNC, siSCD1, siIRE1α, or siXBP1 for 24 h were infected with CSFV (MOI = 1). At 24 hpi, cells were digested with 0.25% trypsin, centrifuged at 1,000 *g* for 5 min, and the supernatant was discarded. Triglyceride content was measured using a commercial detection kit. (**B**) PK-15 cells infected with CSFV (MOI = 1) were treated with the indicated inhibitors for 24 h. Triglyceride content was measured using a commercial detection kit. (C through E) PK-15 cells transfected with siNC, siSCD1, siIRE1α, or siXBP1 for 24 h were infected with CSFV (MOI = 1). At 24 hpi, cells were fixed and stained with rabbit anti-SCD1, anti-IRE1α, anti-XBP1 antibodies, mouse anti-dsRNA monoclonal antibody, or BODIPY 493/503 for confocal microscopy. Scale bars = 10 µm. (**F**) Integrated density of LDs from panels (C through E) was analyzed using ImageJ 7.0 and GraphPad Prism 9 software. (G through J) PK-15 cells infected with CSFV (MOI = 1) were treated with the indicated inhibitors for 24 h. Cells were then fixed and stained with rabbit anti-SCD1 antibody, mouse anti-dsRNA monoclonal antibody, or BODIPY for confocal microscopy. Scale bars = 10 µm. (**K**) Integrated density of LDs from panels (G through J) was analyzed using ImageJ 7.0 and GraphPad Prism 9 software. Data are presented as the mean ± SD from three independent experiments. *P < 0.05, **P < 0.01, ***P < 0.001, ****P < 0.0001.

The primary function of SCD1 is to catalyze the synthesis of unsaturated fatty acids. Oleic acid, a critical small-molecule lipid, is a key component of TG synthesis and is essential for flavivirus infection (36). We hypothesized that intracellular SCD1 expression was positively correlated with TG content. Moreover, LDs, critical intracellular lipid storage organelles (37), provide raw materials and energy needed for viral replication (38), with intracellular TG serving as an essential component of LDs (39). Therefore, we further hypothesized that variations in TG content may affect intracellular LD levels. First, cells were transfected with siRNA targeting SCD1, IRE1α, or XBP1 and subsequently infected with CSFV. At 24 hpi, the cells were harvested, and intracellular TG content was measured. The results demonstrated that intracellular TG content was significantly reduced compared to the control group when endogenous SCD1, IRE1α, or XBP1 expression was knocked down (Fig. 7A). Furthermore, infected cells were treated for 24 h with SCD1 inhibitors (A939572, MF-438, and SCD1 inhibitor-4) and IRE1α/XBP1 inhibitors (GSK2850163 and 4µ8c), harvested, and analyzed for intracellular TG content. The results indicated that, except for MF-438, which showed no significant effects on TG content, treatment with A939572 or SCD1 inhibitor-4 resulted in significant reductions, as did IRE1α and XBP1 inhibitors, compared to the control group (Fig. 7B). Moreover, the changes in LD content were observed using confocal microscopy, which revealed a decrease in intracellular LD content when the expression of endogenous SCD1/IRE1α/XBP1 was reduced compared to the control group (Fig. 7C through F). Similarly, treatment with A939572 and IRE1α/XBP1 inhibitors (GSK2850163 and 4µ8c) significantly reduced LD levels (Fig. 7G through K), supporting a positive correlation between intracellular SCD1 content and TG and LD levels. To summarize, the IRE1α/XBP1 signaling pathway induced by CSFV infection orchestrates SCD1 synthesis, subsequently influencing levels of downstream TG and LD.

## DISCUSSION

Currently, vaccination is the primary strategy for CSFV prevention and control. However, covert and persistent infections of CSFV remain prevalent in clinical settings, causing substantial losses to the livestock industry (40–42). Identifying effective antiviral targets and developing specific therapeutic agents are crucial for the prevention, control, and eventual eradication of CSF. Viruses exploit lipid metabolism through several pathways during infection: (i) directly utilizing lipid metabolism for replication (43), (ii) increasing the activity or quantity of lipid metabolism-related enzymes to facilitate progeny virus production (44); and (iii) utilizing intermediates, such as ATP, for vital viral processes (45). In this study, a library of 96 lipid metabolism-related compounds was screened using IFA, identifying 14 inhibitors with inhibition rates exceeding 60%. Among the various compounds examined, three specific inhibitors of SCD1 have gained considerable focus, emphasizing the essential function of SCD1 in the replication process of CSFV. SCD1, a rate-limiting enzyme, catalyzes the synthesis of oleic acid and palmitoleic acid from stearoyl-CoA and palmitoyl-CoA, facilitating the production of MUFAs from saturated fatty acids (46). Unsaturated fatty acids are essential for viral replication, making enzymes in this pathway promising targets for anti-CSFV drug development. Our data demonstrated that SCD1 inhibitors (A939572, MF-438, and SCD1 inhibitor-4) effectively suppressed CSFV replication. Furthermore, our findings revealed that SCD1 inhibitors suppressed the replication of other *Flaviviridae* family members. Additionally, these inhibitors elucidated inhibitory effects against the DNA virus PRV, suggesting that SCD1-targeting inhibitors were potential broad-spectrum antiviral agents. Our study further substantiated that SCD1 inhibitors did not impact CSFV adsorption or internalization but primarily influenced the later stages of the viral life cycle. During CSFV/JEV infection, SCD1 protein expression initially increased, peaked at 24 hpi, and subsequently decreased. Previous studies have shown that SCD1 transcript, protein, and enzyme activity levels increase during the early stages of viral replication when viral RNA and protein levels are low. However, SCD1 mRNA, protein, and activity exhibit high levels during the early stages of viral replication due to the limited production of viral RNA and protein (12). This observation aligns with our findings. Furthermore, overexpression of SCD1 promoted CSFV replication. Consistently, knockdown of endogenous SCD1 significantly suppressed CSFV replication, indicating a positive correlation between SCD1 protein and viral replication.

**Fig 8:**
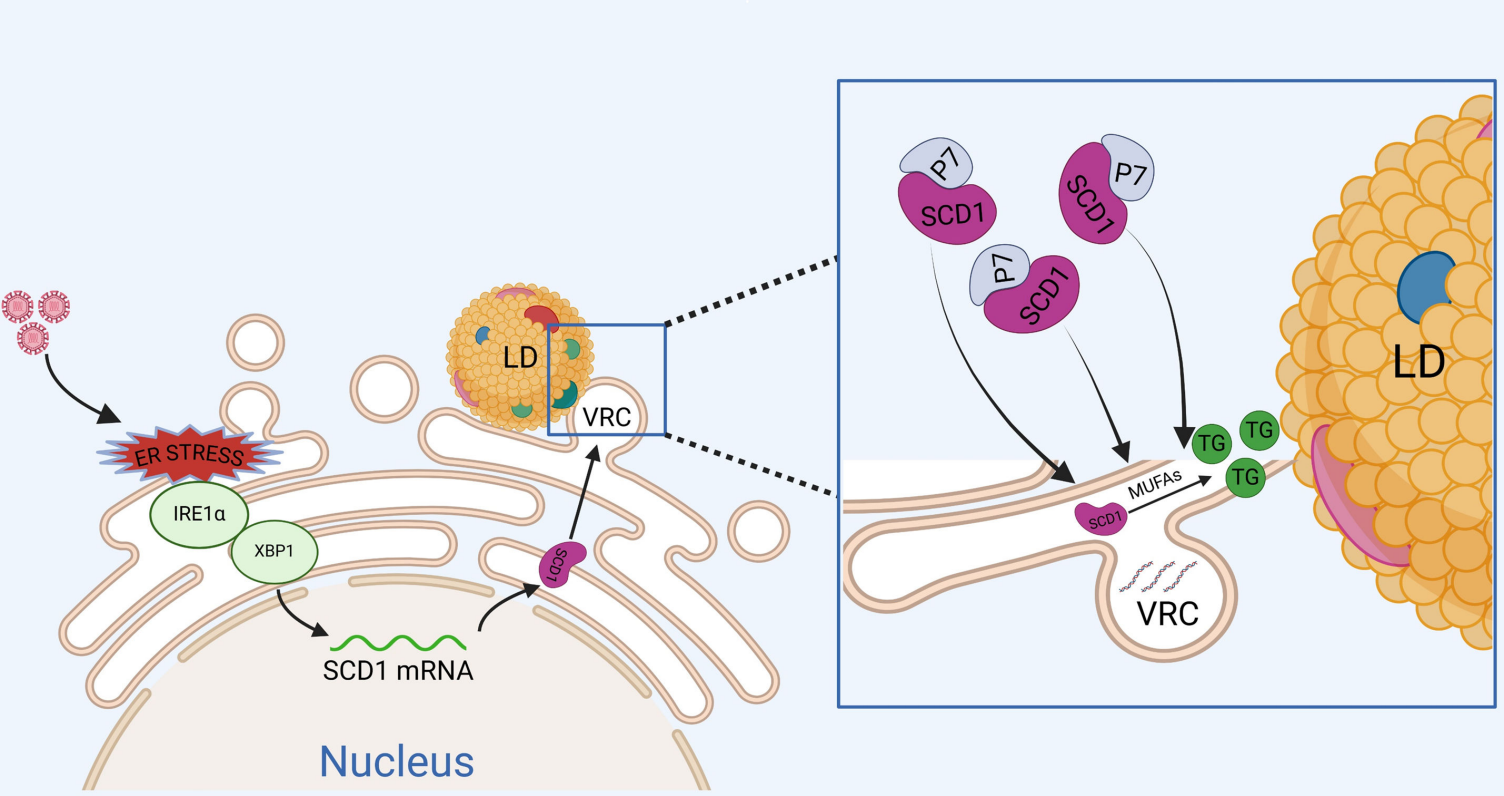
Schematic model depicting the IRE1α/XBP1 pathways and p7 regulating SCD1 during CSFV infection. The replication of CSFV is regulated by the endoplasmic reticulum stress pathway IRE1α/XBP1, which controls the synthesis of SCD1; the IRE1α/XBP1/SCD1 pathway regulates the synthesis of TGs and LDs, thereby affecting CSFV replication. Furthermore, the non-structural protein p7 of CSFV interacts with SCD1 in the endoplasmic reticulum and hijacks SCD1 to the CSFV VRC to promote viral replication.

Moreover, SCD1 was downregulated using inhibitors or siRNA for a duration of 24 h, after which the cells were inoculated with CSFV, followed by inoculation with CSFV (MOI = 1). Oleic acid or palmitoleic acid, the primary products of SCD1, was added in 2% Dulbecco’s modified Eagle’s medium (DMEM) for 24 h, which restored CSFV replication, indicating that SCD1-mediated synthesis of unsaturated fatty acids was essential for CSFV infection. Unexpectedly, it was revealed for the first time that CSFV or JEV infection markedly elevates endogenous SCD1 expression. It is hypothesized that virus-induced SCD1 expression promotes CSFV replication. Previous studies have reported that the non-structural proteins NS3 and NS5A of HCV interact with SCD1 (23). Recently, SCD1 was found to interact with the FMDV 2C (25). In this study, we elucidated for the first time that SCD1 interacts with the non-structural protein p7 of CSFV and is recruited to the VRC. Furthermore, the ion channel inhibitor Bit-225 was employed to investigate the role of p7 in recruiting SCD1 to the VRC. Co-localization of SCD1 with the VRC weakened following inhibitor treatment. However, as Bit-225 is primarily an inhibitor of the Vpu protein (47, 48), which blocks the Vpu ion channel, there is no direct evidence for its effect on the p7 ion channel. Thus, our understanding of how Bit-225 disrupts the recruitment of SCD1 to the VRC via p7 remains preliminary, and further direct evidence is required to elucidate this process. Currently, most studies focus on the relationship between SCD1 enzyme activity and viral life cycles, while few explore whether viruses stimulate intracellular pathways to regulate SCD1. Even less attention has been given to whether these pathways affect the synthesis of lipid precursors for monounsaturated fatty acids or their connections to other lipid organelles. A study has demonstrated that CSFV transiently activates the IRE1 pathway during the initial stages of infection, but subsequently downregulates it, likely due to a reduction in cytoplasmic Ca^2+^ levels following viral incubation (49). In alignment with these findings, the results obtained in this study further substantiate that CSFV infection activated the ER stress pathway IRE1α/XBP1, subsequently regulating SCD1 levels. This aligns with previous findings that oleic acid (31), a critical small-molecule lipid, is essential for triglyceride synthesis, including those stored in LDs, which are vital for flavivirus infection. TG and LD levels were measured in CSFV-infected host cells after SCD1 inhibition. The results confirmed that reduced SCD1 levels correspondingly decreased intracellular TG and LD content. Additionally, we investigated whether this process is regulated by the upstream IRE1α/XBP1 pathway of SCD1. The results revealed that inhibition of IRE1α or XBP1 reduced TG and LD levels, indicating that IRE1α/XBP1-regulated SCD1 modulates intracellular TG and LD content. These findings profoundly augment our comprehension of the intricate nexus between ER stress and lipid metabolism (Fig. 8).

In conclusion, our study substantiates the critical role of SCD1 in the replication process of CSFV. As shown in Fig. 8, upon CSFV infection of host cells, ER stress is induced, which regulates SCD1 through the ER stress pathway IRE1α/XBP1, thereby affecting the synthesis of MUFA. This modulation positively impacts TG and LD formation, ultimately enhancing CSFV replication. Furthermore, the CSFV non-structural protein p7 and SCD1 localize to the ER and interact with each other. p7 appropriates SCD1 to the CSFV VRC to facilitate CSFV replication. This schematic representation underscores that SCD1 is instrumental in the assembly of the CSFV replication complex and promotes the replication of CSFV.

## MATERIALS AND METHODS

### Virus, cells, and plasmids

CSFV Shimen strain (GenBank accession number: AF092448), JEV NJ2008 strain (GenBank accession number: GQ918133), PRV SD2019 strain (GenBank accession number: MW805231), and BVDV2 strain (GenBank accession number: MG420995) were kept in the lab. Cells were cultured in DMEM (GIBCO, Invitrogen, CA, USA) supplemented with 10% fetal bovine serum (GIBCO, Invitrogen, CA, USA), 0.2% NaHCO_3_, 100 µg/mL streptomycin, and 100 IU/mL penicillin (GIBCO, Invitrogen, CA, USA). The pFLAG-NS3, -NS4B, -NS5A, -NS5B, -E2, and -Core plasmids were generated by cloning corresponding CSFV genes into the p3×FLAG-CMV-7.1 vector. The plasmid pEGFP-C1-NS4A was generated by cloning corresponding CSFV NS4A truncated mutant genes into the pEGFP-C1 vector. The plasmid pEGFP-N1-p7 was generated by cloning corresponding CSFV p7 truncated mutant genes into the pEGFP-N1 vector. The plasmid pHA-SCD1 was generated by cloning corresponding SCD1 and its truncated mutant genes into the pcDNA3.1-HA vector. The authenticity of each construct was confirmed by DNA sequencing. The primers were listed in Table 1.

**TABLE 1:** Primers used in this study

Primer	Sequence (5´−3´）	Accession	Use
β-Actin-F	CTCCATCATGAAGTGCGACGT	AJ312193	RT-qPCR for detection of β-actin
β-Actin-R	GTGATCTCCTTCTGCATCCTGTC		
CSFV-F	CCTGAGGACCAAACACATGTTG	AF092448	RT-qPCR for detection of CSFV
CSFV-R	CCTGAGGACCAAACACATGTTG		
JEV-F	AGAGCGGGGAAAAAGGTCAT	GQ918133	RT-qPCR for detection of JEV
JEV-R	AGAGCGGGGAAAAAGGTCAT		
BVDV-F	GAGTACAGGGTAGTCGTCAGT	MG420995	RT-qPCR for detection of BVDV
BVDV-R	CTCTGCAGCACCCTATCAGG		
PRV-F	GATGACCTCAACGGCGACCTC	MW805231	qPCR for detection of PRV
PRV-R	GCGAGAAGAGCTGCGAGTGG		
SCD1-F	CTACACAACCACCACTACCATC	NM_213781	RT-qPCR for detection of SCD1
SCD1-R	CGGATGTCTTCTTCCACGTATT		
XBP1-F	TCCGCAGCACTCAGACTACGT	NM_001142836	RT-qPCR for detection of XBP1
XBP1-R	ATGCCCAAGAGGATATCAGACTC		
IRE1α-F	GCGAAGCATGTGCTGAAACA	XM_005668695	RT-qPCR for detection of IRE1α
IRE1α-R	TATCCGGTCACTCACGTCCT		
pSCD1-F	GGAATTCATGCCGGCCCACTTG	NM_213781	Plasmid construct for pHA- SCD1
pSCD1-R	CCTCGAGTCAGCCACTCTTGTAGCT		
siSCD1-F	GGAGAAACAUCAUCCUUAUTT	NM_213781	siRNA knockdown for SCD1
siSCD1-R	AUAAGGAUGAUGUUUCUCCTT		
siXBP1-F	CAAGCUGGAAGCCAUUAAUTT	NM_001142836	siRNA knockdown for XBP1
siXBP1-R	AUUAAUGGCUUCCAGCUUGTT		
siIRE1α-F	CGGGCCAUGAGAAAUAAGATT	XM_005668695	siRNA knockdown for IRE1α
siIRE1α-R	UCUUAUUUCUCAUGGCCCGTT		

### Cell viability assay

Cells were seeded in a 96-well plate, and once the cell density reached 80%–90%, the compounds were diluted with 2% DMEM to different concentrations and maintained for 24 h. The cytotoxic effects of the compounds on cells were assessed using the Cell Counting Kit-8 (CCK-8, Absin Bioscience Inc., Shanghai, China). After incubation at 37°C for 1–4 h, the absorbance of the reagent was measured using a fluorescence microplate reader at λ 450 nm.

### Screening of lipid metabolism-targeted compounds

Lipid metabolism-targeted compound library was purchased from MedChem Express (MCE, NJ, USA) and stored as 10 mM stock solutions in dimethyl sulfoxide (DMSO) at −80°C until use. Cells were treated separately with the compounds (2 µM compound or DMSO) after infection with CSFV (MOI of 1). At 24 hpi, cells were harvested and subjected to indirect IFA. Fluorescence intensity was measured using ImageJ 7.0 software (ImageJ, MD, USA). The inhibition rate of each compound was normalized to the same volume of the DMSO-treated control group. Each assay was performed in duplicate. In this screening, compounds with a 60% inhibition rate of CSFV replication were selected.

### Binding and entry assay

Cells were pretreated with nontoxic concentrations of compounds at 37°C for 1 h and inoculated with CSFV (MOI of 10) at 4°C for 1 h to allow virus attachment without internalization. Subsequently, cells were washed three times with cold phosphate-buffered saline (PBS) before viral RNA was extracted and quantified by RT-qPCR (binding). The culture medium was replaced with fresh serum-free DMEM, and cells were subsequently shifted to 37°C with 5% CO_2_ to allow virus internalization. After 1 h, cells were washed with citrate buffer solution (pH 3) to remove the noninternalized virions on the surfaces of cells and then washed three times with cold PBS before viral RNA was extracted and quantified by RT-qPCR (entry).

### Time-of-addition assay

Cells were pretreated for 12 h with the compound before the virus was inoculated to the cells (12 h pretreatment). Alternatively, the compound was added only during 1 h of infection, when most of the virus entry and fusion occurred (1 h entry), or after the 1 h of infection (after entry). For the remainder of the infection, cells were pretreated for 12 h and the compound remained throughout the 24 h of infection (throughout). After incubation for 24 h, cells were harvested for RT-qPCR and Western blotting.

### Reverse transcription real-time PCR

Total RNA was extracted from infected cells using TRIzol reagent (Invitrogen, CA, USA), and reverse-transcribed to cDNA using reverse transcription reagent (R222; Vazyme, Nanjing, China). Relative expression levels of target mRNA were determined by RT-qPCR using SYBR qPCR Master Mix (Q511; Vazyme, Nanjing, China). The expression levels for these genes were normalized to that of β-actin, and relative gene expression levels were calculated using the relative standard curve 2^−ΔΔCt^ method. The primers were listed in Table 1.

### Western blotting

Cells were harvested and lysed in radioimmunoprecipitation assay lysis buffer (R0020; Solarbio, Beijing, China) for 15–20 min at 4°C. Lysates were clarified by centrifugation at 12,000 *g* for 10 min at 4°C, and the supernatant was then mixed with 5 × SDS loading buffer. Proteins in the lysates were separated by SDS-PAGE, transferred to nitrocellulose membranes, and probed with the indicated antibodies. β-Actin was used as a loading control. All the antibodies were listed in Table 2. To determine indicated protein levels, the grayscale analysis was performed with the corresponding amount of protein/β-actin by ImageJ 7.0 software.

**TABLE 2:** Antibodies used in this study

Antibody	Name	Supplier	Catalog no.
Flag	Anti-Flag M2 antibody	Sigma-Aldrich	F1804
HA	Anti-HA antibody mouse MAb	Sigma-Aldrich	H3663
β-Actin	β-Actin (C4) antibody	Santa Cruz	sc-47778
dsRNA	Mouse dsRNA antibody (J2)	Scicons	1001050
GFP	Rabbit anti GFP-Tag mAb	Abclonal	AE078
XBP1	XBP1 rabbit pAb	Abclonal	A1731
SCD1	SCD1 antibody	Affinity	DF13253
IRE1α	IRE1; ERN1 polyclonal antibody	Proteintech	27528-1-AP

### Plasmids and siRNA transfection

Cells grown to 60%–80% confluence cell culture plates were transfected with specified plasmids using jetPRIME transfection reagent (Polyplus, France) as per the manufacturer’s protocol. For RNA interference, cells were transfected with siRNA using Lipofectamine RNAiMAX (Invitrogen, CA, USA) according to the manufacturer’s instructions. All the siRNA duplexes were listed in Table 1, which were designed and synthesized by GenePharma Co., Ltd. (Shanghai, China). Cells were infected with CSFV (MOI of 1) and harvested for Western blotting at 24 hpi.

### Co-immunoprecipitation

Cells were transfected with indicated plasmids for 24 h and lysed in lysis buffer (50 mM Tris-HCl pH7.5; 150 mM NaCl; 1% Triton X-100; 1 mM EDTA; 1 mM phenylmethylsulfonyl fluoride [PMSF]) for 30 min at 4°C. The lysate was collected and centrifuged at 12,000 *g* for 5 min at 4°C to obtain the supernatant (whole-cell lysate). A 100 µL aliquot of the supernatant was removed from all samples for later use. The remaining lysate was incubated with GFP/HA antibody rotating overnight, then the samples were incubated with agarose beads (Alpalifebio, Shenzhen, China) and continuously rotated for 4–6 h at 4°C. The agarose beads were collected by centrifugation at 3,000 *g* for 5 min at 4°C and then resuspended in 2× SDS loading buffer for Western blotting.

### Confocal microscopy and IFA

Cells grown on coverslip dishes or 12-well plates were fixed with 4% paraformaldehyde (PFA) for 30 min at room temperature and permeabilized with 0.1% Triton X-100 (Sigma, USA) for 15 min at room temperature. Cells were incubated with first primary antibodies overnight at 4°C and then incubated with fluorescent-labeled secondary antibodies for 1 h at 37°C. The cell nuclei were stained with 4',6-diamidino-2-phenylindole (DAPI) (C1005, Beyotime, Shanghai, China) for 10 min. For confocal microscopy, cells were scanned by Nikon A1 confocal microscopy (Nikon, Japan), and co-localization coefficients were calculated using ImageJ 7.0 software. For IFA, cells on 96-well plates were examined under a Zeiss LSM700 confocal microscopy (Zeiss, Germany).

### Triglyceride detection

The triglyceride content of the cells was measured using the triglyceride assay kit (Nanjing Jiancheng Bioengineering Institute; Nanjing, China). After three washes with cold PBS, cells in a 60-mm dish were digested with 0.25% trypsin and subsequently collected into a centrifuge tube. The collected cells were centrifuged at 1 ,000 *g* for 5 min, and the supernatant was discarded. Then cells were lysed with 2% Triton X-100 (Sigma, USA) for 30 min. Then, the lysate reacted with the working solution at 37°C for 10 min and was detected at λ 500 nm.

### LDs and Golgi staining

Cells grown on coverslip dishes or 12-well plates were fixed with 4% PFA for 30 min at room temperature. Subsequently, cells were incubated with first primary antibodies overnight at 4°C and then incubated with fluorescent-labeled secondary antibodies for 1 h at 37°C. After that, cells were incubated with indicated ER/Golgi-Tracker Red (C1041S/C1043, Beyotime, Shanghai, China) for 30 min at 37°C. The cell nuclei were stained with DAPI (C1005, Beyotime, Shanghai, China) for 10 min. For confocal microscopy, cells were scanned by Nikon A1 confocal microscope (Nikon, Japan), and co-localization coefficients were calculated using ImageJ 7.0 software. For IFA, cells cultured on 96-well plates were examined under a Zeiss LSM700 confocal microscopy (Zeiss, Germany).

### Statistical analysis

All data were presented as means ± standard deviations as indicated. Student’s *t*-test was used to compare the data from pairs of treated and untreated groups. Statistical significance is indicated by asterisks in the figures. All statistical analyses and calculations were performed using Prism 6 (GraphPad Software Inc., La Jolla, CA).

## Data Availability

All relevant data are within the article.
